# Direct-to-Consumer Recruitment Methods via Traditional and Social Media to Aid in Research Accrual for Clinical Trials for Rare Diseases: Comparative Analysis Study

**DOI:** 10.2196/39262

**Published:** 2023-03-14

**Authors:** Janelle Applequist, Cristina Burroughs, Peter A Merkel, Marc Rothenberg, Bruce Trapnell, Robert Desnick, Mustafa Sahin, Jeffrey Krischer

**Affiliations:** 1 Zimmerman School of Advertising & Mass Communications University of South Florida Tampa, FL United States; 2 Health Informatics Institute University of South Florida Tampa, FL United States; 3 Division of Rheumatology, Department of Medicine University of Pennsylvania Philadelphia, PA United States; 4 Division of Clinical Epidemiology, Department of Biostatistics, Epidemiology, and Informatics University of Pennsylvania Philadelphia, PA United States; 5 Allergy and Immunology, Department of Pediatrics Cincinnati Children's Hospital Medical Center Cincinnati, OH United States; 6 Department of Internal Medicine University of Cincinnati College of Medicine Cincinnati, OH United States; 7 Icahn School of Medicine at Mount Sinai New York, NY United States; 8 Rosamund Stone Zander Translational Neuroscience Center Boston Children's Hospital Boston, MA United States

**Keywords:** direct-to-consumer advertising, clinical trial recruitment, clinical trial accrual, research recruitment, research participant recruitment, social media recruitment, web-based recruitment, patient-centered research, rare diseases, clinical trial

## Abstract

**Background:**

Recruitment into clinical trials is a challenging process, with as many as 40% of studies failing to meet their target sample sizes. The principles of direct-to-consumer (DTC) advertising rely upon novel marketing strategies. The ability to reach expansive audiences in the web-based realm presents a unique opportunity for researchers to overcome various barriers to enrollment in clinical trials. Research has investigated the use of individual web-based platforms to aid in recruitment and accrual into trials; however, a gap in the literature exists, whereby multiple mass communication platforms have yet to be investigated across a range of clinical trials.

**Objective:**

There is a need to better understand how individual factors combine to collectively influence trial recruitment. We aimed to test whether DTC recruitment of potentially eligible study participants via social media platforms (eg, Facebook [Meta Platforms Inc] and Twitter [Twitter Inc]) was an effective strategy or whether this acted as an enhancement to traditional (eg, email via contact registries) recruitment strategies through established clinical research sites.

**Methods:**

This study tested multiple DTC web-based recruitment efforts (Facebook, Twitter, email, and patient advocacy group [PAG] involvement) across 6 national and international research studies from 5 rare disease consortia. Targeted social media messaging, social media management software, and individual study websites with prescreening questions were used in the Protocol for Increasing Accrual Using Social Media (PRISM).

**Results:**

In total, 1465 PRISM website referrals occurred across all 6 studies. Organic (unpaid) Facebook posts (676/1465, 46.14%) and Rare Diseases Clinical Research Network patient contact registry emails (461/1465, 31.47%) represented the most successful forms of engagement. PRISM was successful in accumulating a 40.1% (136/339) lead generation (those who screened positive and consented to share their contact information to be contacted by a clinical site coordinator). Despite the large number of leads generated from PRISM recruitment efforts, the number of patients who were subsequently enrolled in studies was low. Across 6 studies, 3 participants were ultimately enrolled, meaning that 97.8% (133/136) of leads dropped off.

**Conclusions:**

The results indicate that although accrual results were low, this is consistent with previously documented challenges of studying populations with rare diseases. Targeted messaging integrated throughout the recruitment process (eg, referral, lead, and accrual) remains an area for further research. Key elements to consider include structuring the communicative workflow in such a way that PAG involvement is central to the process, with clinical site coordinators actively involved after an individual consents to share their contact information. Customized approaches are needed for each population and research study, with observational studies best suited for social media recruitment. As evidenced by lead generation, results suggest that web-based recruitment efforts, coupled with targeted messaging and PAG partnerships, have the potential to *supplement* clinical trial accrual.

## Introduction

### Background

It is widely accepted that the enrollment of individuals into clinical trials can be an arduous process, even in instances where a specialized infrastructure for recruitment is in place. Estimates show that approximately half (40%) of National Clinical Trial Network trials fail to complete accrual [1]. Approximately one-fifth of randomized controlled trials close without meeting at least 85% of their target accrual [2].

The COVID-19 pandemic added a unique layer of additional challenges to the conduct of clinical research. Many non–COVID-19 studies had to consider novel ways of adapting their processes to recruit and gain the consent of new patients, especially through web-based systems [3]. There was, and remains, an urgency for research activities to continue while keeping study staff and participants safe. Therefore, emphasis on best practices for study recruitment, web-based study information, and web-based consent became more important than ever for the medical field.

The pharmaceutical industry has notably established direct-to-consumer (DTC) advertising as an effective format for informing individuals of treatment options for various diagnoses [4]. Such DTC messaging practices focus on directly targeting patient populations rather than through health care professionals [5-9]. The principles of DTC advertising rely upon novel marketing strategies that can be applied to other situations, specifically, recruitment for clinical trials. Social media and the web remain 2 areas for further exploration. The ability to reach expansive audiences in the web-based realm presents a unique opportunity for researchers to overcome various barriers to enrollment in clinical trials.

Progress has been made in developing strategies aimed at improving the recruitment process for clinical trials; however, research emphasizing the use of social media often focuses on the use of one platform for recruitment (eg, Facebook [Meta Platforms Inc]) [10]. Previous research addressing the incorporation of social media recruitment strategies has documented a mixture of successes and failures. Positive findings associated with using social media as a recruitment tool include its cost-effectiveness when compared with more traditional forms of media (radio, television, or print), its ability to penetrate more global communities, the flexibility associated with being able to control and track recruitment efforts, and its ability to improve participant selection for hard-to-reach demographics [11-13]. Primary challenges associated with such techniques include the considerable amount of personnel time required for managing content, participant confusion surrounding the discernment between legitimate and fraudulent information on the internet, roadblocks investigators encounter with ethical review boards when using web-based recruitment strategies, and this type of recruitment yielding few participants [10,14-16].

In addition, existing research on social media strategies often emphasizes key health areas (health promotion, cancers, circulatory system diseases, and mental health) or specific populations (few studies focused on underserved or minority groups), limiting the generalizability of results regarding web-based recruitment efforts for clinical trials [17]. To the best of our knowledge, none of the existing studies examine the use of multiple recruitment strategies using targeted messaging across different study populations. A more cohesive, multipronged approach is needed to determine what types of participant groups, target populations, and studies are best suited for various DTC recruitment efforts. There is a need for research to test the effectiveness of alternative recruitment strategies with targeted messaging efforts.

### Consideration of the Population With Rare Diseases

Patients with rare diseases are expressing an all-time high level of interest in clinical trial participation, with nearly 9 out of 10 individuals (88%) saying they would consider using an investigational treatment [18]. However, the population with rare diseases presents a unique challenge when considering accrual into clinical trials, as these patients comprise a group that is often out of reach of the already few geographically limited clinical centers involved in recruiting participants for various studies [19,20]. For many rare diseases, only a few specialists exist nationwide or worldwide, so patients often travel long distances to access treatment [18]. Rare diseases are defined as those that affect <200,000 individuals in the United States, with >6000 rare disease diagnoses recognized [21]. Thus, the total number of people with at least 1 rare disease is large; however, owing to the low incidence of individual rare disease diagnoses, accruing sufficient number of study participants for research in rare diseases poses a major challenge. In a 2019 cross-sectional analysis of 659 rare disease randomized clinical trials, 30.2% of trials (199/659) were closed owing to the lack of patient accrual (64/659, 32.1%), the most common reason cited for trial noncompletion [22].

The Rare Diseases Clinical Research Network (RDCRN) is an international clinical research initiative of the National Institute of Health’s Office of Rare Diseases Research and the National Center for Advancing Translational Sciences. The RDCRN manages research studies of >200 rare diseases. Centralized coordination for these studies was housed at the Data Management and Coordinating Center at the University of South Florida from 2003 to 2019, where all data and protocol activity for >100 of the studies managed by the 21 rare disease consortia that comprise RDCRN were coordinated using electronic data capture systems.

### Research Objectives

There is a need for research to better understand how individual factors combine to collectively influence trial recruitment. This study tested whether DTC recruitment of potentially eligible study participants via social media platforms (eg, Facebook [Meta Platforms Inc] and Twitter [Twitter Inc]) was an effective strategy or whether this process acted as an enhancement to the already-existing, traditional (eg, email via contact registries) recruitment strategies through established clinical research sites. This study focused on determining which patient populations and study designs lent themselves to the use of targeted recruitment messaging. The specific objectives of this study were as follows:

1. Compare *referral percentages* of the following individuals:
  - Those who clicked on the respective study website, in which case we identified the source that lead them there (eg, Facebook; defined as a *referral*)
  - Those who completed the *prescreener* with pass or fail rates (questions on study inclusion and exclusion criteria)
  - Those who consented to share their contact information and completed the registration form (defined as a *lead*)
  - Those who enrolled in the respective RDCRN study (*enrollment* is defined as the process of registering and screening an eligible participant who has consented and agreed to participate in the study)
2. Compare percentages for differences across strata for 6 varying studies (based on the type of study, demographic characteristics of the *leads* generated, and characteristics of the disease)

Previous research documented a need for clinical trials to use a variety of recruitment methods in addition to traditional advertising measures for optimal results, including the more recent consideration of DTC practices emphasizing social marketing strategies and support from patient advocacy groups (PAGs) [13,23]. To investigate the aforementioned objectives, an integrated system for the implementation of various recruitment practices was developed, titled Protocol for Increasing Accrual Using Social Media (PRISM). PRISM was a web-based, patient-centric tool for identifying potential participants and referring them to more study information, prescreening, and enrollment. Designed as a comprehensive framework for the implementation of various recruitment methods, PRISM was developed to capture the analytics of various targeted messaging efforts; lead individuals interested in a clinical research study to a website where they could learn more information; and motivate individuals to complete prescreeners after which they could potentially be deemed eligible for study participation, consent to share their contact information, and then be contacted by a study team member for subsequent study enrollment.

PRISM was designed to test the contribution that DTC recruitment practices can make to clinical studies through the use of various marketing techniques. Namely, we sought to investigate the various touchpoints in the aforementioned recruitment workflow to determine the role that social media can play. This study’s use of DTC included the integration of social media (Facebook and Twitter) and traditional, mass communicative recruitment techniques (patient contact registries and email) and the incorporation of PAG support. The implementation of PRISM and its framework relied upon a patient-centric process, wherein pilot research determined the best practices for recruitment message language, framing, and presentation [24].

## Methods

### Time Period Analyzed

All data were collected and analyzed from 2018 to 2020, predating the 2020 wave of the COVID-19 pandemic. Social media recruitment took place, and individual study websites were live from December 1, 2018, to June 30, 2019, meaning that this study looks at a 7-month sample of data.

### Ethics Board Review

All PRISM recruitment methods and procedures received expedited Institutional Review Board approval (Pro00034181). Electronic informed consent was obtained from interested participants before a prescreening survey could be administered on each study's website. Standard Health Insurance Portability and Accountability Act language was included with the informed consent to ensure participants that their personal health information would only be shared with PRISM staff in a deidentified format for research purposes and with clinical site coordinators who would contact them if they passed the prescreener, thus being deemed eligible to participate in the study. Contact information and screening question responses were the only information shared with study site staff. Therefore, sites participating in PRISM did not need individual IRB approval, as one site was responsible for the recruitment tactics. If participants decided to enroll in a study post-recruitment procedures, they relied upon each site's individual IRB for that study's protocol.

### Study Selection

To test DTC recruitment methods across various patient populations and study types, a comprehensive review of all the existing research studies led by the RDCRN to be considered for inclusion in PRISM was conducted. Six research studies from 5 rare disease consortia were selected (Table 1). It was not required that the PRISM study be registered on ClinicalTrials.gov; however, the individual RDCRN studies chosen for comparative purposes were registered (ClinicalTrials.gov NCT02108860, NCT02939573, NCT03531996, NCT03118674, NCT02991807, and NCT02523118 [25-30]). Studies were purposively chosen to include a variety of research designs, investigational agents, levels of necessary participant involvement, disease characteristics, and target population demographics. These 5 consortia spanned a range of these parameters.

**Table 1:** Representative Rare Diseases Clinical Research Network protocols included in Protocol for Increasing Accrual Using Social Media.

Protocol	Consortium	Clinical site locations	Target accrual	Study type	Intervention type	Disease of interest	Age range in protocol	Disease demographics	Disease status at enrollment	Disease incidence or prevalence
Abatacept (CTLA4-Ig) for the Treatment of Relapsing, Non-Severe, Granulomatosis With Polyangiitis (ABROGATE; ClinicalTrials.gov NCT02108860)	VCRC^a^ 5527	United States, Canada, United Kingdom, Ireland, and Germany	66	Interventional RCT^b^— phase III	Double blinded Placebo-controlled investigational agent	GPA^c^ or Wegener’s granulomatosis	≥15 years	Average age of onset is 45 years, but the disease may manifest at any age. It affects both males and females.	Mild disease flare—active disease at enrollment	Prevalence in the United States is estimated to be 3.0 per 100,000.
A Randomized, Multicenter Study for Isolated Skin Vasculitis (ARAMIS; ClinicalTrials.gov NCT02939573)	VCRC 5562	United States and Canada	90	Interventional sequential multiple assignment RCT	3 standard of care medications	Skin or cutaneous vasculitis and IgA^d^ vasculitis or HSP^e^	≥18 years	Cutaneous vasculitis affects both sexes and all ages. It affects adults more often than children, and 90% of pediatric cases will have IgA vasculitis.	Active disease at enrollment	Overall annual incidence of cutaneous vasculitis ranges from 39.6 to 59.8 per million.
Longitudinal Evaluation of Autoimmune Pulmonary Alveolar Proteinosis (LongPAP; ClinicalTrials.gov NCT03531996)	RLD^f^ 5712	United States	100	Longitudinal and observational	None	PAP^g^	All ages	Median age at the time of diagnosis is 39 years; most patients are men, and 72% of those with this condition have a history of smoking.	Active disease or remission (no major disease activity)	Prevalence is estimated to be 0.37 per 100,000 persons.
Newer Direct-Acting Anti-Viral Agents as Sole Therapy of Porphyria Cutanea Tarda in Subjects With Chronic Hepatitis C (ClinicalTrials.gov NCT03118674)	PC^h^ 7210	United States	49	Interventional	Open label One arm	PCT^i^	≥18 years	Disease manifests in adulthood. Men are more affected than women.	PCT with chronic hepatitis C	Prevalence is estimated to be approximately 1 in 25,000.
A Randomized Double-Blind Controlled Trial of Everolimus in Individuals With PTEN Mutations (ClinicalTrials.gov NCT02991807)	DSC^j^ 7904	United States	40	Interventional—phase 1 or 2	Placebo controlled Investigational agent	PTEN^k^ PHTS^l^ or PTEN	5-45 years	It is a genetic condition that affects both men and women and may present in either childhood or adulthood.	Outpatients with PTEN genetic mutation	Overall prevalence for PHTS has not been well established. Prevalence of Cowden syndrome is estimated to be 1 in 200,000.
A Prospective, Multicenter Study to Compare and Validate Endoscopic, Histologic, Molecular, and Patient-Reported Outcomes in Pediatric and Adult Patients With Eosinophilic Esophagitis, Gastritis, and Colitis (ClinicalTrials.gov NCT02523118)	CEGIR^m^ 7801	United States	1050	Observational	None	EoG^n^ or EoC^o^	≥3 years	It affects both males and females. Onset may occur in children or adults.	Active disease at enrollment	Estimated prevalence is <1 in 20,000 persons each in the United States.

^a^VCRC: Vasculitis Clinical Research Consortium.

^b^RCT: randomized controlled trial.

^c^GPA: granulomatosis with polyangiitis.

^d^IgA: immunoglobulin A.

^e^HSP: Henoch Schönlein purpura.

^f^RLD: Rare Lung Disease Consortium.

^g^PAP: pulmonary alveolar proteinosis.

^h^PC: Porphyrias Consortium.

^i^PCT: porphyria cutanea tarda.

^j^DSC: Developmental Synaptopathies Consortium.

^k^PTEN: phosphatase and tensin homolog.

^l^PHTS phosphatase and tensin homolog hamartoma tumor syndrome.

^m^CEGIR: Consortium of Eosinophilic Gastrointestinal Disease Researchers.

^n^EoG: eosinophilic gastritis.

^o^EoC: eosinophilic colitis.

### Targeted Messaging

**Figure 1:**
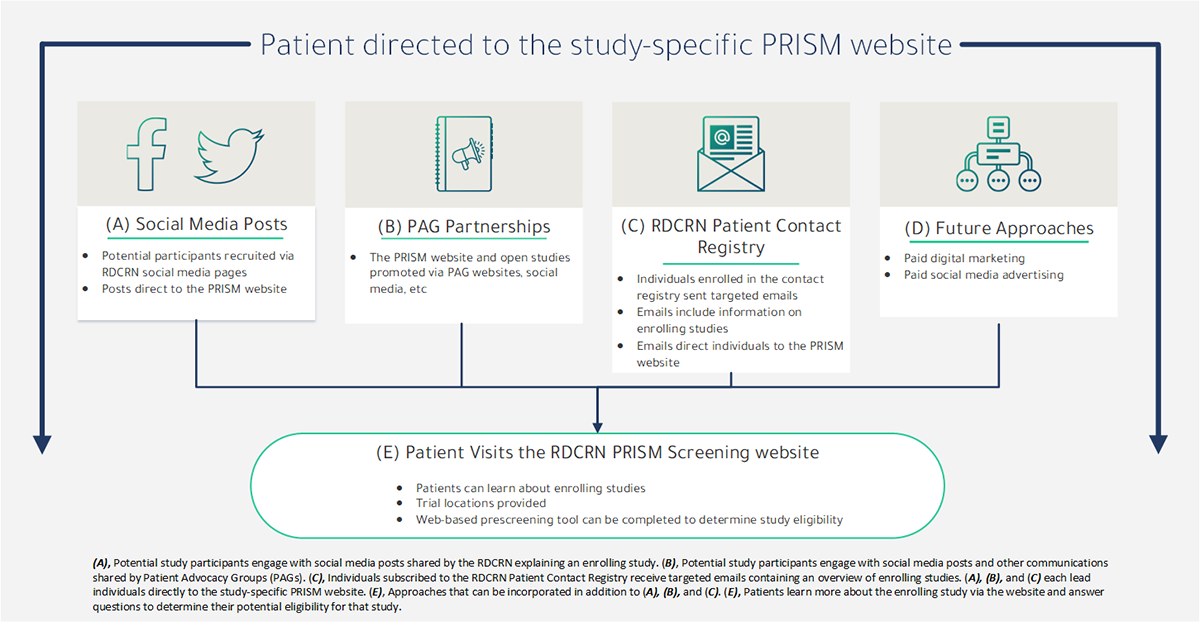
Protocol for Increasing Accrual Using Social Media (PRISM) recruitment workflow. PAG: patient advocacy group; RDCRN: Rare Diseases Clinical Research Network; SEO: search engine optimization.

PRISM was a multistep process (Figure 1) that began with a variety of DTC recruitment efforts initiated by the RDCRN. Recruitment efforts used included study-specific email blasts sent to those enrolled in the respective RDCRN consortium for updates, organic social media posts (content posted for free) shared by the RDCRN’s Facebook and Twitter accounts, and paid social media posts via Facebook.

Although the studies tested in PRISM did use traditional methods of recruitment (eg, physician referrals and direct outreach), our study’s focus was on DTC measures to find additional approaches to recruitment into studies on rare diseases to best harness the potential for social media to effectively aid in recruitment.

A health communication scholar designed all DTC recruitment posts to ensure that targeted messaging and proper health literacy levels were incorporated. Previous pilot research for PRISM conducted in-depth interviews with representatives from each target population to determine how best to tailor messages used for recruitment purposes [31]. Targeted messages were designed for each study featured in PRISM, with different design elements incorporated based on the target population and various considerations that needed to be made based on the rare disease itself according to feedback from PAGs and patient interviews. For example, messages for the Consortium of Eosinophilic Gastrointestinal Disease Researchers (CEGIR) study did not feature stock photos of families during mealtime, as this was often seen as a challenging time for individuals and caregivers in the target population, given that food allergies are a common occurrence with such eosinophilic gastrointestinal diseases.

All targeted messaging approaches adopted in PRISM, including recruitment email messages, social media posts, individual study landing pages, and prescreening questions, were developed using the Step Approach to Message Design and Testing, a conceptual framework that guides the iterative, reflexive design of communication messages by assessing the individual characteristics of the target audience, characteristics of individual messages, emotional and cognitive responses to individual messages, and acceptance or rejection of the messages [31]. The Step Approach to Message Design and Testing, designed to develop and evaluate various types of persuasive health messages, allowed for the pilot testing of all materials with patients with rare diseases and PAG representatives, with feedback incorporated at each phase to ensure that the messages resonated with their intended audiences.

All the diseases studied had PAGs of various sizes, usually comprising families of patients affected by the disease who advocate for individuals, treatments, and research [32]. PAG representatives were also asked to review all recruitment messages designed by the RDCRN. Their feedback was subsequently incorporated into all message design phases before recruitment messages were finalized. As the RDCRN’s existing social media accounts were ultimately used to share the recruitment messages, an important component of PRISM involved incorporating PAGs into the social media recruitment phases. PAGs agreed to share respective study recruitment content through their existing social media networks, allowing the recruitment messages of RDCRN to gain greater exposure. PAGs asked that text be added to recruitment content clarifying that their sharing of a post from the RDCRN was not an endorsement of the respective study. The RDCRN then supplied each PAG with a personalized social media kit to make it as easy as possible for them to share the recruitment content. Each social media kit included ready-made posts that could be shared with audiences to maximize recruitment efforts.

### Social Media Management Software

Subscription to a third-party social media management software was purchased for the purposes of PRISM. As multiple DTC recruitment messages were shared via the RDCRN’s existing social media accounts, it was found necessary and helpful to use a service that permitted for the scheduling of posts at various times. Sprout Social (Sprout Social Inc) was used to schedule all posts and to provide analytics on individual posts. The RDCRN’s existing social media accounts were used to share all DTC messages created for the PRISM study recruitment. Social media efforts related to PRISM began on December 1, 2018, and ended on June 30, 2019. Analytics measured via Sprout Social included the number of individual posts, likes, shares, impressions (number of times a post was displayed on someone’s screen for the first time), engagement (number of times an action was performed in relation to the post, such as sharing and clicking), average daily reach (average number of people who viewed the post at least once), and clicks. Sprout Social also permitted for the tracking of organic versus paid posts, whereby organic posts were uploaded by the RDCRN at no cost, and a small advertising budget of US $585 was used toward paid Facebook advertisements over the 7-month data collection period.

### Individual Study Websites With Web-Based Prescreening

PRISM represented a novel approach beyond its recruitment efforts. Traditional clinical trial enrollment efforts require that the study staff contact a prospective participant, explain the study to them, review their eligibility, obtain their informed consent, and ultimately enroll them in the trial. If a participant clicked on a recruitment message (via social media or otherwise), they were directed to the study-specific website.

In an effort to simultaneously inform patients of study information and prescreen those who may have been eligible to participate, a foundational base website was created. This template was then used to create 6 individual websites, 1 for each PRISM study, whereby individuals could learn more about the study, qualification criteria, funding source or sources, type of intervention, length of commitment, potential compensation, etc.

The design of each website (presented in Figure S1 of Multimedia Appendix 1) was informed by in-depth interviews and user testing completed with patients from each rare disease population before the launch of PRISM. Appropriate health literacy levels were incorporated into each iteration of the individual website design.

While on the respective study landing website page, individuals could click “get started now,” after which they could answer a series of ≤10 questions designed to act as a prescreening mechanism for the research study. Research has suggested that in instances where social media is used as a recruitment mechanism, prescreening questions should be used to bolster study enrollment percentages [33]. The prescreening questions (presented in Figure S2 of Multimedia Appendix 1) developed for each site were based on individual study inclusion and exclusion criteria and were designed by a health communication scholar in collaboration with the research study team.

**Figure 2:**
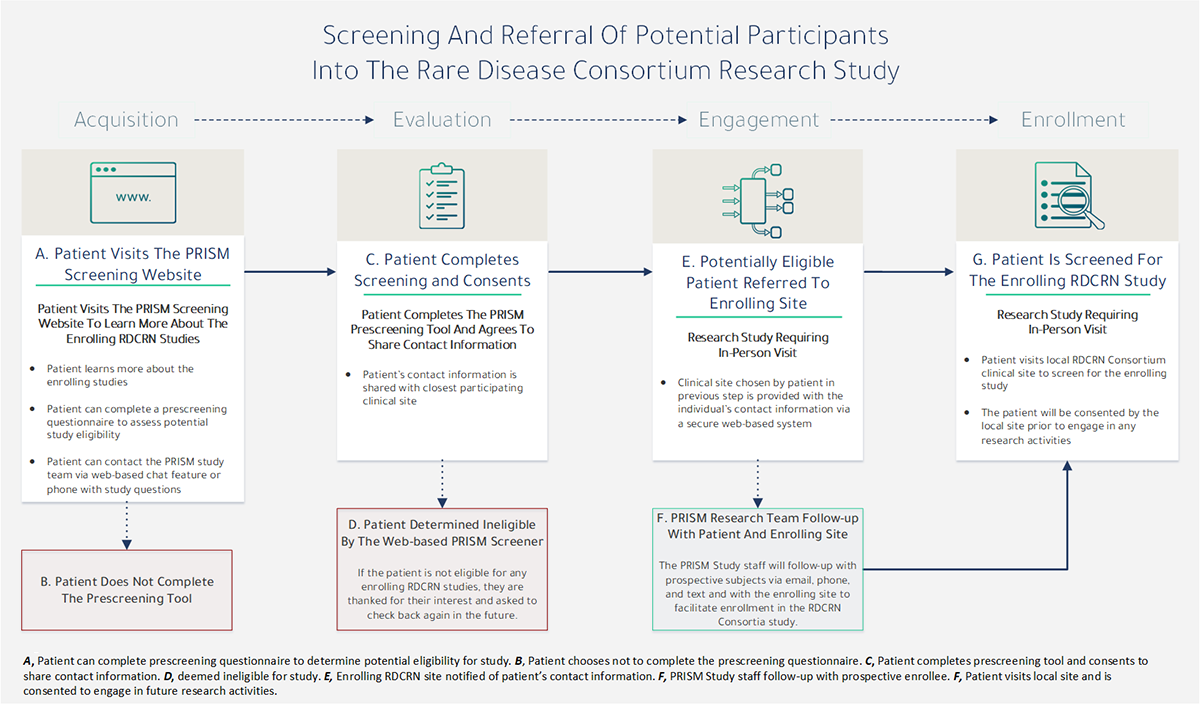
Protocol for Increasing Accrual Using Social Media (PRISM) postrecruitment workflow. RDCRN: Rare Diseases Clinical Research Network.

If an individual was deemed eligible upon completing the prescreening questions, a new web page would appear asking for their consent to share their information with the closest study site of their choice. Upon consenting, the patient would then enter their name, contact information, and address. Once the address was entered, a geocoding process would then provide the patient with a Google Maps (Google LLC) list of the study sites geographically closest to their location. This process (Figure 2) served as the primary mechanism for recruitment, whereby individual study sites received the contact information of individuals who passed the prescreening round. Therefore, participants could not be fully screened until they were contacted by the local site coordinator, at which point they could decide to consent using the local site’s institutional review board– or ethics board–approved informed consent form and enroll in the respective study.

## Results

### Overview

The following outcomes were measured for each of the 6 PRISM studies: (1) overall PRISM referrals, (2) web-based prescreener pass or fail rates and number of PRISM leads (those deemed eligible to participate following the prescreener and consented to share their contact information with the corresponding clinical site), and (3) comparison of the outcomes 1 and 2 in light of the differences across studies (type of study, type of drug, demographic features of the target population, and disease characteristics). In addition, (4) the number of RDCRN study enrollments were considered.

**Figure 3:**
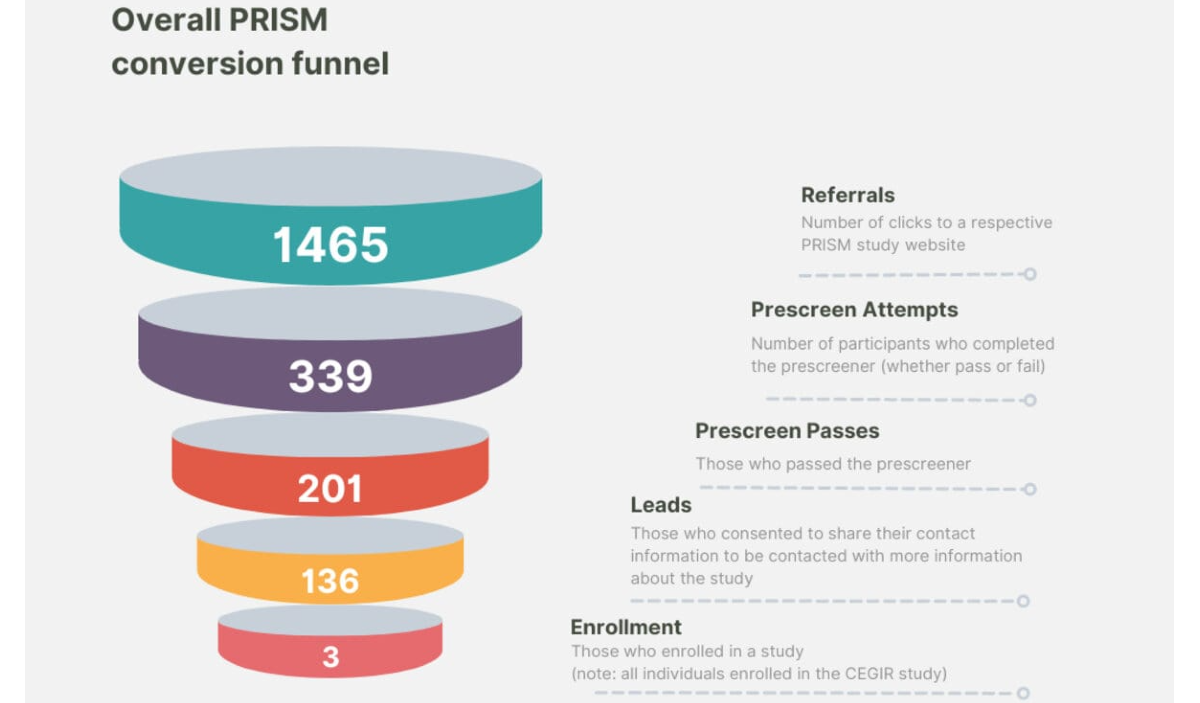
Overall Protocol for Increasing Accrual Using Social Media (PRISM) conversion rates. CEGIR: Consortium of Eosinophilic Gastrointestinal Disease Researchers.

A summary of results is provided in Figure 3 and Table 2.

**Table 2:** Protocol for Increasing Accrual Using Social Media conversion rates by protocol (N=1465 for all protocols combined).

Protocol, consortium, and recruitment type				Website users				Users who consented		
				n (%)		N		n (%)		N
**Abatacept (CTLA4-Ig) for the Treatment of Relapsing, Non-Severe, Granulomatosis With Polyangiitis (ABROGATE)**										
	**VCRC^a^ 5527**									
		Organic Facebook	18 (7.5)		239		0 (0)		18	
		Paid Facebook advertising	39 (16.3)		239		0 (0)		39	
		Organic Twitter	26 (10.9)		239		0 (0)		26	
		Other web-based recruitment	37 (15.5)		239		0 (0)		37	
		RDCRN^b^ patient contact registry	117 (49.0)		239		8 (6.8)		117	
		PAG^c^ outreach	2 (0.8)		239		0 (0)		2	
**A Randomized, Multicenter Study for Isolated Skin Vasculitis (ARAMIS)**										
	**VCRC 5562**									
		Organic Facebook	74 (57.3)		129		0 (0)		74	
		Paid Facebook advertising	10 (7.8)		129		0 (0)		10	
		Organic Twitter	29 (22.5)		129		0 (0)		29	
		Other web-based recruitment	4 (3.1)		129		0 (0)		4	
		RDCRN patient contact registry	12 (9.3)		129		1 (8.3)		12	
		PAG outreach	0 (0)		129		0 (0)		0	
**Longitudinal Evaluation of Autoimmune Pulmonary Alveolar Proteinosis (LongPAP)**										
	**RLD^d^ 5712**									
		Organic Facebook	82 (38)		216		0 (0)		8	
		Paid Facebook advertising	10 (4.6)		216		0 (0)		10	
		Organic Twitter	29 (13.4)		216		0 (0)		29	
		Other web-based recruitment	3 (1.4)		216		0 (0)		3	
		RDCRN patient contact registry	92 (42.6)		216		33 (35.9)		92	
		PAG outreach	0 (0)		216		0 (0)		0	
**Newer Direct-Acting Anti-Viral Agents as Sole Therapy of Porphyria Cutanea Tarda in Subjects With Chronic Hepatitis C**										
	**PC^e^ 7210**									
		Organic Facebook	49 (48.5)		101		0 (0)		49	
		Paid Facebook advertising	3 (3)		101		0 (0)		3	
		Organic Twitter	15 (14.9)		101		0 (0)		15	
		Other web-based recruitment	0 (0)		101		0 (0)		0	
		RDCRN patient contact registry	32 (31.6)		101		7 (21.9)		32	
		PAG outreach	2 (2)		101		0 (0)		2	
**A Randomized Double-Blind Controlled Trial of Everolimus in Individuals With PTEN Mutations**										
	**DSC^f^ 7904**									
		Organic Facebook	31 (11.4)		273		0 (0)		31	
		Paid Facebook advertising	28 (10.2)		273		1 (3.6)		28	
		Organic Twitter	40 (14.7)		273		3 (7.5)		40	
		Other web-based recruitment	14 (5.1)		273		0 (0)		14	
		RDCRN patient contact registry	160 (58.6)		273		16 (10)		160	
		PAG outreach	0 (0)		273		0 (0)		0	
**A Prospective, Multicenter Study to Compare and Validate Endoscopic, Histologic, Molecular, and Patient-Reported Outcomes in Pediatric and Adult Patients With Eosinophilic Esophagitis, Gastritis, and Colitis**										
	**CEGIR^g^ 7801**									
		Organic Facebook	422 (83.2)		507		46 (10.9)		422	
		Paid Facebook advertising	7 (1.4)		507		0 (0)		7	
		Organic Twitter	21 (4.1)		507		1 (4.8)		21	
		Other web-based recruitment	9 (1.8)		507		1 (11.1)		9	
		RDCRN patient contact registry	48 (9.5)		507		19 (39.6)		48	
		PAG outreach	0 (0)		507		0 (0)		0	

^a^VCRC: Vasculitis Clinical Research Consortium.

^b^RDCRN: Rare Diseases Clinical Research Network.

^c^PAG: patient advocacy group.

^d^RLD: Rare Lung Disease Consortium.

^e^PC: Porphyrias Consortium.

^f^DSC: Developmental Synaptopathies Consortium.

^g^CEGIR: Consortium of Eosinophilic Gastrointestinal Disease Researchers.

### Overall PRISM Referrals

PRISM referrals were DTC messages that a user clicked (eg, Facebook posts and email links), which led them to a study’s website. In Table 3, referral sources are organized by study, and the types of recruitment referrals are shown.

An organic (nonpaid) Facebook post originating from the main RDCRN account meant that the post was published for free, using the RDCRN’s existing network of approximately 2000 web-based followers. The paid Facebook posts or advertisements originating from the main RDCRN account incorporated Facebook Insights (eg, gender and interests) to help use target market strategies to increase recruitment efforts for maximum message distribution. Similar to an organic Facebook post, an organic Twitter post relied upon the existing network of the RDCRN’s followers (approximately 6500 at the time of data collection). Paid Twitter posts were not used for this exploratory study. *Other online recruitment posts* included links to the PRISM website posted on ClinicalTrials.gov, the consortium public website pages, and the clinical study site website pages (if applicable). In addition, the RDCRN patient contact registry, a database containing clinical data about individuals who have specific conditions, was used to send emails to potentially eligible patients [34]. Finally, PAG outreach acted as a point of referral by sending the RDCRN-provided emails to their existing contact registries.

The CEGIR study had the highest level of organic Facebook reach, representing 62.4% (422/676) of the total organic Facebook referrals across all studies (Table 2). The Developmental Synaptopathies Consortium (DSC) study received the greatest number of referrals via the RDCRN patient contact registry emails, representing 34.7% (160/461) of these total emails in the 6-study sample.

A total of 1465 PRISM website referrals occurred across all 6 studies. Organic (not paid) Facebook posts (676/1465, 46.1%) represented the most successful form of engagement, followed by RDCRN patient contact registry emails (461/1465, 31.5%; Table 4).

**Table 3:** Protocol for Increasing Accrual Using Social Media referrals (N=1465 for all protocols combined).

Protocol, consortium, and recruitment type				Users who visited an individual study website, n (%)
**Abatacept (CTLA4-Ig) for the Treatment of Relapsing, Non-Severe, Granulomatosis With Polyangiitis (ABROGATE)**				
	**VCRC^a^ 5527 (n=239)**			
		Organic Facebook	18 (7.5)	
		Paid Facebook advertising	39 (16.3)	
		Organic Twitter	26 (10.9)	
		Other web-based recruitment	37 (15.5)	
		RDCRN^b^ patient contact registry	117 (49)	
		PAG^c^ outreach	2 (0.8)	
**A Randomized, Multicenter Study for Isolated Skin Vasculitis (ARAMIS)**				
	**VCRC 5562 (n=129)**			
		Organic Facebook	74 (57.3)	
		Paid Facebook advertising	10 (7.8)	
		Organic Twitter	29 (22.5)	
		Other web-based recruitment	4 (3.1)	
		RDCRN patient contact registry	12 (9.3)	
		PAG outreach	0 (0)	
**Longitudinal Evaluation of Autoimmune Pulmonary Alveolar Proteinosis (LongPAP)**				
	**RLD^d^ 5712 (n=216)**			
		Organic Facebook	82 (38)	
		Paid Facebook advertising	10 (4.6)	
		Organic Twitter	29 (13.4)	
		Other web-based recruitment	3 (1.4)	
		RDCRN patient contact registry	92 (42.6)	
		PAG outreach	0 (0)	
**Newer Direct-Acting Anti-Viral Agents as Sole Therapy of Porphyria Cutanea Tarda in Subjects With Chronic Hepatitis C**				
	**PC^e^ 7210 (n=101)**			
		Organic Facebook	49 (48.5)	
		Paid Facebook advertising	3 (3)	
		Organic Twitter	15 (14.9)	
		Other web-based recruitment	0 (0)	
		RDCRN patient contact registry	32 (31.6)	
		PAG outreach	2 (2)	
**A Randomized Double-Blind Controlled Trial of Everolimus in Individuals With PTEN Mutations**				
	**DSC^f^ 7904 (n=273)**			
		Organic Facebook	31 (11.4)	
		Paid Facebook advertising	28 (10.2)	
		Organic Twitter	40 (14.7)	
		Other web-based recruitment	14 (5.1)	
		RDCRN patient contact registry	160 (58.6)	
		PAG outreach	0 (0)	
**A Prospective, Multicenter Study to Compare and Validate Endoscopic, Histologic, Molecular, and Patient-Reported Outcomes in Pediatric and Adult Patients With Eosinophilic Esophagitis, Gastritis, and Colitis**				
	**CEGIR^g^ 7801 (n=507)**			
		Organic Facebook	422 (83.2)	
		Paid Facebook advertising	7 (1.4)	
		Organic Twitter	21 (4.1)	
		Other web-based recruitment	9 (1.8)	
		RDCRN patient contact registry	48 (9.5)	
		PAG outreach	0 (0)	

^a^VCRC: Vasculitis Clinical Research Consortium.

^b^RDCRN: Rare Diseases Clinical Research Network.

^c^PAG: patient advocacy group.

^d^RLD: Rare Lung Disease Consortium.

^e^PC: Porphyrias Consortium.

^f^DSC: Developmental Synaptopathies Consortium.

^g^CEGIR: Consortium of Eosinophilic Gastrointestinal Disease Researchers.

**Table 4:** Overall Protocol for Increasing Accrual Using Social Media referrals by recruitment type (N=1465).

Recruitment type	Users who visited an individual study website, n (%)
Organic Facebook	676 (46.1)
Twitter	160 (10.9)
Paid Facebook advertising	97 (6.6)
Other web-based recruitment	67 (4.6)
RDCRN^a^ patient contact registry	461 (31.5)
PAG^b^ outreach	4 (0.3)

^a^RDCRN: Rare Diseases Clinical Research Network.

^b^PAG: patient advocacy group.

### Web-Based Prescreener Pass or Fail Rates and the Number of PRISM Leads

Across the 6 studies, 339 prescreen attempts were completed. Respondents “passed” the attempt if they were deemed potentially eligible to participate in the study (upon further review or consultation with a clinical study site coordinator). They “failed” the attempt if they did not meet the study’s eligibility criteria as determined by the prescreening questions. Prescreen pass rates accounted for 59.3% (201/339) of the total cases, but these individuals then needed to consent to share their contact information for prescreen pass to then become classified as a PRISM lead.

A high level of variance in the pass or fail rates for the prescreening questions was observed across studies (Table 5). For example, 80% (48/60) of prescreen attempts for the Vasculitis Clinical Research Consortium 5527 study failed. However, 80.3% (94/117) of respondents passed the prescreening process for the CEGIR protocol.

Overall, 40.1% (136/339) of all prescreen attempts resulted in leads. However, some studies resulted in much higher lead generation than their counterparts. The CEGIR (67/136, 49.3%), Rare Lung Disease Consortium (RLD; 33/136, 24.2%), and DSC (20/136, 14.7%) studies comprised 88.2% (120/136) of all the leads.

**Table 5:** Protocol for Increasing Accrual Using Social Media (PRISM) screening summary by protocol.

Protocol	Consortium	Respondents passing prescreen (n=202), n (%)	Respondents failing prescreen (n=138), n (%)	Conversion rate (%)^a^	PRISM leads (n=136), n (%)
Abatacept (CTLA4-Ig) for the Treatment of Relapsing, Non-Severe, Granulomatosis With Polyangiitis (ABROGATE)	VCRC^b^ 5527	12 (5.9)	48 (34.8)	66.67	8 (5.9)
A Randomized, Multicenter Study for Isolated Skin Vasculitis (ARAMIS)	VCRC 5562	4 (2)	12 (8.7)	25	1 (0.7)
Longitudinal Evaluation of Autoimmune Pulmonary Alveolar Proteinosis (LongPAP)	RLD^c^ 5712	49 (24.3)	5 (3.6)	67.35	33 (24.3)
Newer Direct-Acting Anti-Viral Agents as Sole Therapy of Porphyria Cutanea Tarda in Subjects With Chronic Hepatitis C	PC^d^ 7210	9 (4.5)	17 (12.3)	77.78	7 (5.1)
A Randomized Double-Blind Controlled Trial of Everolimus in Individuals With PTEN Mutations	DSC^e^ 7904	94 (46.3)	23 (16.7)	71.28	67 (49.3)
A Prospective, Multicenter Study to Compare and Validate Endoscopic, Histologic, Molecular, and Patient-Reported Outcomes in Pediatric and Adult Patients With Eosinophilic Esophagitis, Gastritis, and Colitis	CEGIR^f^ 7801	34 (16.8)	33 (23.9)	58.82	20 (14.7)

^a^Total conversion rate is 67.37%.

^b^VCRC: Vasculitis Clinical Research Consortium.

^c^RLD: Rare Lung Disease Consortium.

^d^PC: Porphyrias Consortium.

^e^DSC: Developmental Synaptopathies Consortium.

^f^CEGIR: Consortium of Eosinophilic Gastrointestinal Disease Researchers.

### Differences Across RDCRN Studies

The CEGIR, RLD, and DSC studies represented the most successful studies in terms of PRISM referrals and subsequent lead generation. However, these studies have variations in terms of study and intervention types (Table 1). CEGIR and RLD are observational or longitudinal studies, whereas DSC is an interventional placebo-controlled trial. All the studies that were most successful in web-based referrals were open to pediatric populations or participants of all ages.

The demographic features of the target population and disease characteristics for each RDCRN study are summarized in Table 1.

The demographic features of the overall leads generated for all the studies are presented in Tables S1-S5 in Multimedia Appendix 1. Individuals identifying as not of Hispanic or Latino origin were most likely to click on recruitment materials. Although there was variance in terms of the age of the participants who engaged with web-based recruitment, those in their 30s were the most responsive.

### Number of RDCRN Study Enrollments

Despite the large number of leads generated from PRISM recruitment efforts, the number of patients who were subsequently enrolled in an RDCRN study was small (Table 2). Across all 6 studies, 3 participants ultimately enrolled. All 3 enrolled in the CEGIR study. This means that 97.8% (133/136) of PRISM leads dropped off.

Overall, the 2 recruitment types with the best response rates were the RDCRN contact registry (18.22% conversion rate) and organic Facebook posts (6.8% conversion rate; Table 6). There was variance in the number of email blasts sent to each prospective study pool based on the size of the rare disease population and the number of people in each consortia contact registry (as shown in Table S5 in Multimedia Appendix 1).

**Table 6:** Overall Protocol for Increasing Accrual Using Social Media conversion rates by recruitment type (N=1465).

Recruitment type	Users who visited an individual study website (N=1465), n (%)	Users who consented	
		n (%)	N
Organic Facebook	676 (46.1)	46 (6.8)	676
Twitter	160 (10.9)	4 (2.5)	160
Paid Facebook advertising	97 (6.6)	1 (1)	97
Other web-based recruitment	67 (4.6)	1 (1.5)	67
RDCRN^a^ patient contact registry	461 (31.5)	84 (18.2)	461
PAG^b^ outreach	4 (0.3)	0 (0)	4

^a^RDCRN: Rare Diseases Clinical Research Network.

^b^PAG: patient advocacy group.

## Discussion

### Principal Findings

This exploratory research suggests that observational studies may be best suited for web-based recruitment efforts when aiming for ultimate enrollment. Although 97.8% (133/136) of PRISM leads dropped off, it is important to note that these enrollment rates, known in advertising and marketing as click-through rates (CTRs), are consistent with industry averages. In the United States, the average CTR for emails is 3.3%, the average CTR for Twitter is 1.64%, and the average CTR for Facebook is 0.89% [35-37]. In addition, when considering the challenges associated with the population with rare diseases in terms of research accrual, it is important to note that these enrollment figures are not entirely surprising. Small sample sizes are inevitable in rare disease research given the number of individuals impacted. Researchers have documented the challenges of working with small data in the context of rare diseases, with some even discussing an American Statistical Association editorial’s suggestion to move away from the use of statistical significance when describing results with *P* values <.05 [38,39]. In light of this, although a 2.2% conversion rate is low, it is important to keep in mind the context of research in rare diseases, which already struggles with the challenges of clinical trial accrual given the disease population sizes (compared with other, more highly diagnosed diseases) [40,41]. The studies used in PRISM are in various stages of completion. It is common for trials in the RDCRN to take years to complete recruitment given the rarity of the diseases and that trials engage only a subset of patients with a given disease. In addition, the COVID-19 pandemic led to a major disruption of all clinical trials. Therefore, we were most interested in exploring various touchpoints throughout the recruitment process, and we understand that messaging cannot always have an instantaneous impact—more integrated marketing efforts may be necessary.

Although PRISM was not a successful mechanism for increasing *enrollment* into RDCRN studies, a notable finding of this study is the importance of the *process* of novel recruitment methods. Low accrual in populations with rare diseases is an ongoing challenge, one that must be studied alongside proper advertising and marketing metrics (key performance indicators). PRISM presented a viable opportunity, as evidenced by the considerable lead initiation (136/339, 40.1%) resulting from *organic* social media posts and contact registry emails. Indeed, when accounting for the aforementioned CTRs used in advertising and marketing, PRISM did account for substantial lead generation and met the average industry-standard conversion targets. Therefore, it is possible that the dropout at the time of study enrollment may have been attributable to other trial factors, such as strict inclusion or exclusion criteria. In cases where an overly restrictive trial design limits the pool of participants, no amount of targeted social media can solve accrual challenges; however, in the case of rare disease research, potential participants have very limited access to opportunities, and social media presents a platform for sharing trials with the target audiences [42].

This study demonstrates the importance of web-based research recruitment using the existing, ready-made social media followings and contact registries to capitalize on built-in audiences. However, it is important to note that there is not a *one-size-fits-all* approach toward success in social media recruitment across multiple rare disease diagnoses. Given the high variability in the number of affected individuals across diseases, it is probable that individualized, fine-tuned approaches are needed for each population and each research study.

In addition, potential study participants demonstrated their ability to understand and correctly appraise the eligibility criteria through the completion of prescreening questions on the study websites. Lead generation was one of the most successful aspects of PRISM. The considerable dropout rate following lead generation (generation of potentially eligible participants who passed the prescreening questions and consented to share their contact information with the corresponding clinical site coordinator) demonstrates a critical area for reflection. One such area is geography; enrollment was limited to sites that may not have been convenient for the generated leads. Another area for reflection is the overall communicative workflow. PRISM may have seen greater success if the final step of the enrollment process (where clinical site coordinators contacted potentially eligible participants and got them enrolled in the study) was given the time and attention it required. In the case of contact registry emails, it is important to note that although much of the current work on web-based recruitment methods revolves around innovative technologies, many patients, as evidenced by this study, are most comfortable using basic forms of web-based communication. Thus, email marketing should not be forgotten as a mechanism to be fine-tuned for web-based clinical trial recruitment. Ensuring that the messaging itself (eg, health literacy levels, use of pixelated images, and appropriate subject lines that catch a patient’s attention) is patient centered is an important consideration for web-based recruitment strategies.

Several aspects of PRISM were successful and could be adapted for future efforts to enhance recruitment. First, social media and email DTC marketing efforts were successful in mobilizing certain individuals to the RDCRN study web pages but not underrepresented minorities. Additional research will be necessary to determine whether web-based recruitment efforts could work in these populations. Next, 40.1% (136/339) of all prescreen attempts resulted in leads, with an overall lead conversion rate of 67.37%, indicating that PRISM presented a notable opportunity for novel study enrollment with the use of technology. However, there was great variation across study types in terms of pass or fail rates. The CEGIR study recruitment audience was present throughout PRISM as an active, engaged audience. This study, which performed the best in terms of PRISM recruitment efforts, represented the RDCRN study with the least perceivable risk, as it was an observational study with no intervention. This means that there may be a study type best suited for web-based recruitment. CEGIR’s PAGs, who agreed to share the RDCRN’s recruitment posts via social media, had an already-existing strong social media network following, presenting a rationale as to why this protocol outperformed others in terms of lead generation.

In the case of the CEGIR study, the PAG’s willingness to share the RDCRN’s recruitment message on its own social media platform with its followers meant that an already-established, engaged, and targeted audience was provided in advance, which likely influenced the chances of the recruitment being effective. A highly engaged CEGIR social media audience influenced the likelihood that individuals saw these recruitment posts and clicked for more information. However, this study’s target population was individuals aged ≥3 years, meaning that parents needed to feel comfortable enrolling their children in the study, which influenced the type of recruitment messaging designed.

Therefore, PAG engagement and capitalizing off of existing web-based networks are key to successful web-based recruitment efforts. An important component of marketing is garnering awareness. For example, we do not expect patients to see an advertisement for a clinical trial and immediately enroll in the study. Instead, advertising helps to build what is referred to as the “drip drip drip” of awareness, whereby individuals are exposed to a variety of messages so that when they are faced with a call to action (to enroll in a study), they are more likely to be persuaded to act on the intended behavior [43]. Thus, the success of the RDCRN contact registry may be attributed to the awareness created by its previous email blasts and its preestablished network. However, future research may want to separate the use of contact registries from social media recruitment efforts to delineate the degree of effectiveness for each recruitment approach.

Our results only showed that this process *can* work but that the dropout occurred at the clinical site or individual contact level, where human contact was necessary to get someone to enroll in the study. Future research must further investigate this point of the workflow, as this breakdown in communication arguably has the power to negate all the recruitment steps beforehand if a patient is not followed up with to be enrolled following their consent to share contact information.

It is also important to note that trial design may be a factor in dropout rates. Strict inclusion or exclusion criteria can be attributable to low accrual rates, and although meeting the goals for research is certainly paramount, no amount of targeted social media will be able to act as a magic wand to solve overly restrictive trial designs.

It will be important for scholars to address the ways in which consumer privacy concerns and changes in data tracking across various social media platforms inevitably alter the landscape for this type of research. As our study did not request consent to share contact information based on geolocation services until a participant passed the prescreener, we could not determine whether an individual had been exposed to more than one recruitment method. To fully capture whether users may have received more than one of our recruitment messages, a customized open-source data integration pipeline would be necessary, meaning that consent would be needed to monitor users’ IP addresses from their initial encounter with the PRISM recruitment messages. However, the ethics surrounding this type of data monitoring pose important questions surrounding patient privacy concerns, particularly surrounding the area of health information [15,44]. Finally, it is important to note that even given the recent changes in privacy settings and advertisement policies (eg, cookie tracking) in Apple Inc and Meta Platforms Inc (Facebook), the techniques described in our study are still applicable, as long as participants consent to sharing their contact information and geolocation data after passing the prescreen. For future research, a pop-up screen may need to be added to study websites asking individuals to consent to the cookies being collected to access their data, which is standard procedure given the changes described.

In addition, it is important to note that because of our informed consent procedures, we did not collect consent to track IP addresses (to have geolocation to share a clinical research site) until *after* an individual had completed the prescreening form. Therefore, we could not align the users for specific studies that screened positive with the recruitment type (eg, Facebook and email) with which they had engaged. However, we could make broader generalizations regarding recruitment types. Future efforts should consider collecting informed consent earlier in the process to increase analytical tracking capabilities. However, this could deter individuals from wanting to complete the prescreening process.

As evidenced by lead generation (Table 5), the results of this study suggest that web-based recruitment efforts, coupled with strategically designed targeted messaging and PAG partnerships, have the potential to help *supplement* clinical trial accrual. However, this is a process that must be fine-tuned, and future research can help better understand the ways in which recruitment efforts can be optimized using web-based technologies. In addition, this study did not address the full potential of social media that could have been realized using a substantial advertising budget (eg, one comparable with the pharmaceutical advertising industry).

### Conclusions

Although this study focused on populations with rare diseases, investigators across many fields have needed to reconfigure their research for the continuity of data collection and the benefit of being able to target previously hard-to-reach populations using the principles of DTC advertising and marketing. The principal results of this study can be applied to various trial settings to leverage technology and increase recruitment efforts. As all 6 protocols chosen for PRISM were in-person studies, its potential impact could have been limited, given the time and travel constraints placed on prospective enrollees. Thus, future research should further investigate the use of this model for web-based studies.

Future research will need to focus on refining each stage of the recruitment and study enrollment processes. Specifically, enhancing the communicative approaches used by the clinical site coordinators after prospective enrollees have consented to sharing their contact information on the web will be integral to the success of such novel methods. In this study, this is where the communication broke down, resulting in individuals being eligible to participate in studies but ultimately not being followed up with enough or in an optimal manner to get them to enroll in the study. It remains unclear exactly why this part of the process dissolved, but future research should further explore this touchpoint.

## Appendix

Multimedia Appendix 1 Supplementary figures of individual study page and prescreening question sample and supplementary tables of Protocol for Increasing Accrual Using Social Media leads based on demographic information.

## Abbreviations

CEGIR: Consortium of Eosinophilic Gastrointestinal Disease Researchers
CTR: click-through rate
DSC: Developmental Synaptopathies Consortium
DTC: direct-to-consumer
PAG: patient advocacy group
PRISM: Protocol for Increasing Accrual Using Social Media
RDCRN: Rare Diseases Clinical Research Network
RLD: Rare Lung Disease Consortium
