# Supplementary material for: Direct-to-Consumer Recruitment Methods via Traditional and Social Media to Aid in Research Accrual for Clinical Trials for Rare Diseases: Comparative Analysis Study
Source: J Med Internet Res. 2023 Mar 14;25:e39262. doi: 10.2196/39262 (PMC10131902; doi:10.2196/39262)
Supplement: Multimedia Appendix 1 [file jmir_v25i1e39262_app1.docx]

This is a Multimedia Appendix to a full manuscript published in the J Med Internet Res. For full copyright and citation information see http://dx.doi.org/10.2196/jmir.39262

Figure S1. PRISM landing page/website.


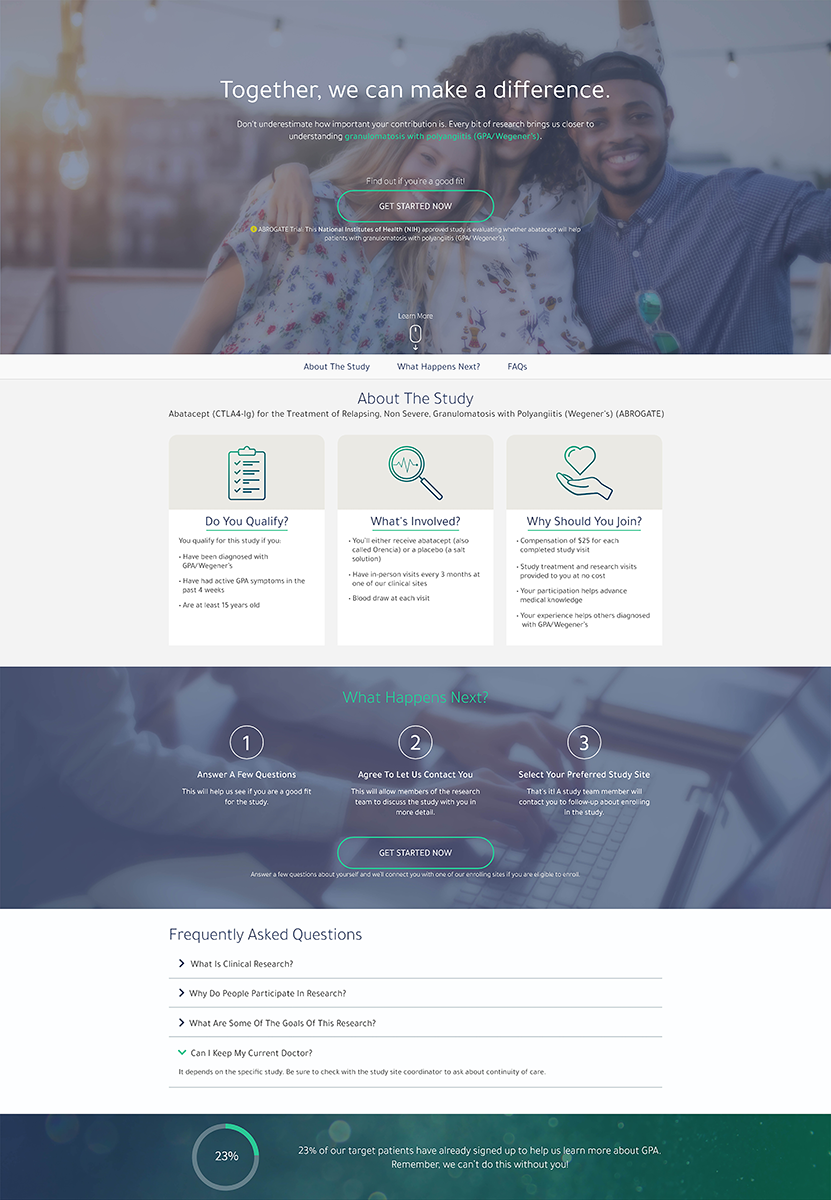


Figure S2. Pre-screening questions for ABROGATE study.


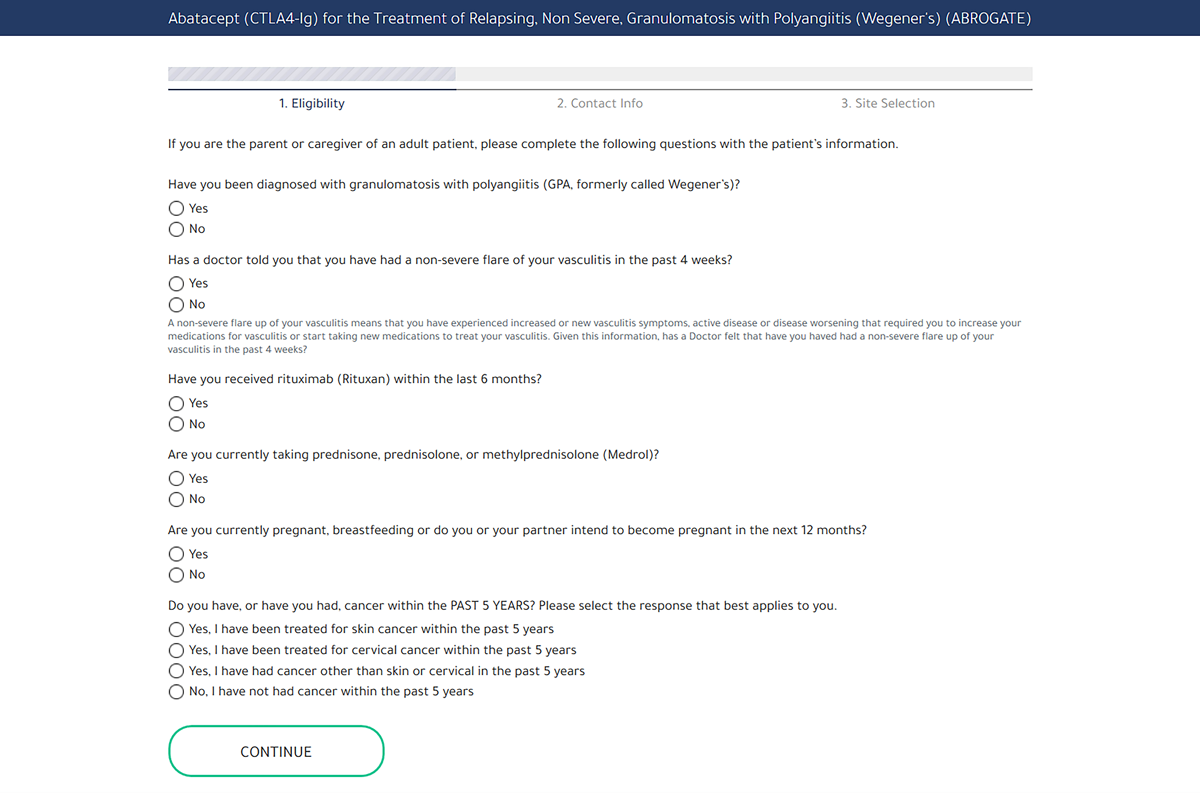


Table S1. Overall PRISM leads by sex, race, and age.

| Gender | Race | Current Age Group | *n* |
| --- | --- | --- | --- |
|  |  |  |  |
| **Female** |  |  |  |
|  | American Indian/Alaskan Native | 41 to 45 | 2 |
|  | Asian | 21 to 25 | 2 |
|  |  | 31 to 35 | 62 |
|  | Black or African American | 6 to 10 | 2 |
|  | Native Hawaiian or Other Pacific Islander | 6 to 10 | 2 |
|  | White | 0 to 5 | 6 |
|  |  | 6 to 10 | 6 |
|  |  | 16 to 20 | 4 |
|  |  | 21 to 25 | 14 |
|  |  | 26 to 30 | 14 |
|  |  | 31 to 35 | 20 |
|  |  | 36 to 40 | 18 |
|  |  | 41 to 45 | 20 |
|  |  | 46 to 50 | 22 |
|  |  | 56 to 60 | 2 |
|  | Unknown or Not Reported | 36 to 40 | 2 |
|  |  | 41 to 45 | 2 |
|  |  | 46 to 50 | 2 |
| **Male** |  |  |  |
|  | Asian | 11 to 15 | 2 |
|  |  | 36 to 40 | 2 |
|  | Black or African American | 46 to 50 | 2 |
|  | White | 0 to 5 | 4 |
|  |  | 6 to 10 | 14 |
|  |  | 11 to 15 | 24 |
|  |  | 16 to 20 | 8 |
|  |  | 21 to 25 | 4 |
|  |  | 26 to 30 | 4 |
|  |  | 31 to 35 | 8 |
|  |  | 36 to 40 | 16 |
|  |  | 41 to 45 | 12 |
|  |  | 46 to 50 | 10 |
|  | Unknown or Not Reported | 26 to 30 | 2 |
|  |  | 36 to 40 | 4 |
|  |  | 56 to 60 | 2 |
|  |  | Missing | 2 |

Table S2. Overall PRISM leads by ethnicity and race.

| Demographic Category | Demographic Subcategory | Males (*n*) | Females (*n*) | Unknown or Not Reported | Total (*N*) |
| --- | --- | --- | --- | --- | --- |
|  |  |  |  |  |  |
| **Ethnicity** |  |  |  |  |  |
|  | Hispanic or Latino Origin | 3 | 8 | 1 | 12 |
|  | Not Hispanic or Latino Origin | 49 | 57 | 3 | 109 |
|  | Unknown or Not Reported | 8 | 6 | 1 | 15 |
| Total |  | *60* | *71* | *5* | *136* |
| **Race** |  |  |  |  |  |
|  | American Indian/Alaskan Native | 0 | 1 | 0 | 1 |
|  | Asian | 2 | 2 | 0 | 4 |
|  | Black or African American | 1 | 1 | 0 | 2 |
|  | More than one Race | 0 | 0 | 0 | 0 |
|  | Native Hawaiian or Other Pacific Islander | 0 | 1 | 0 | 1 |
|  | White | 53 | 63 | 4 | 120 |
|  | Unknown or Not Reported | 4 | 3 | 1 | 8 |
| **Total** |  | *60* | *71* | *5* | *136* |

Table S3. Overall Hispanic or Latino PRISM leads by sex.

| Demographic Category | Demographic Subcategory | Males (*n*) | Females (*n*) | Unknown or Not Reported | Total (*N*) |
| --- | --- | --- | --- | --- | --- |
|  |  |  |  |  |  |
| **Race** |  |  |  |  |  |
|  | American Indian/Alaskan Native | 0 | 1 | 0 | 1 |
|  | Asian | 0 | 0 | 0 | 0 |
|  | Black or African American | 0 | 0 | 0 | 0 |
|  | More than one Race | 0 | 0 | 0 | 0 |
|  | Native Hawaiian or Other Pacific Islander | 0 | 0 | 0 | 0 |
|  | White | 3 | 7 | 1 | 11 |
|  | Unknown or Not Reported | 0 | 0 | 0 | 0 |
| **Total** |  | *3* | *8* | *1* | *12* |

Table S4. Overall PRISM leads by age.

| Age Group | Number of leads (*n*) | Percent of Total Leads |
| --- | --- | --- |
|  |  |  |
| 0 |  |  |
|  | 0 | 0 |
| 0 to 5 |  |  |
|  | 10 | 4% |
| 6 to 10 |  |  |
|  | 24 | 9% |
| 11 to 15 |  |  |
|  | 26 | 10% |
| 16 to 20 |  |  |
|  | 14 | 5% |
| 21 to 25 |  |  |
|  | 20 | 7% |
| 26 to 30 |  |  |
|  | 22 | 8% |
| 31 to 35 |  |  |
|  | 32 | 12% |
| 36 to 40 |  |  |
|  | 44 | 16% |
| 41 to 45 |  |  |
|  | 38 | 14% |
| 46 to 50 |  |  |
|  | 36 | 13% |
| 56 to 60 |  |  |
|  | 4 | 1% |
| Missing |  |  |
|  | 2 | 1% |

Table S5. PRISM email blast summary example.

| Protocol | Consortium | Date Email Blast Sent | Number of Contact Registrants Sent Email |
| --- | --- | --- | --- |
|  |  |  |  |
| **Abatacept (CTLA4-Ig) for the Treatment of Relapsing, Non-Severe, Granulomatosis with Polyangiitis (ABROGATE)** | Vasculitis Clinical Research Consortium (VCRC 5527) | 9/24/18 | 1,546 |
|  |  |  |  |
| **A Randomized, Multicenter Study for Isolated Skin Vasculitis (ARAMIS)** | Vasculitis Clinical Research Consortium (VCRC 5562) | 9/24/18 | 123 |
|  |  |  |  |
| **Longitudinal Evaluation of Autoimmune Pulmonary Alveolar Proteinosis (LongPAP)** | Rare Lung Disease Consortium (RLD 5712) | 10/12/18 | 52 |
|  |  |  |  |
| **Newer Direct-Acting Anti-Viral Agents as Sole Therapy of Porphyria Cutanea Tarda in Subjects with Chronic Hepatitis C** | Porphyrias Consortium (PC 7210) | 9/24/18 | 304 |
|  |  |  |  |
| **A Randomized Double-Blind Controlled Trial of Everolimus in Individuals with PTEN Mutations** | Developmental Synaptopathies Consortium (DSC 7904) | 9/24/18 | 128 |
|  |  |  |  |
| **A Prospective, Multicenter Study to Compare and Validate Endoscopic, Histologic, Molecular, and Patient-Reported Outcomes in Pediatric and Adult Patients with Eosinophilic Esophagitis, Gastritis, and Colitis** | Consortium of Eosinophilic Gastrointestinal Disease Researchers (CEGIR 7801) | 9/24/18 | 233 |
